# Obesity Epidemic—The Underestimated Risk of Endometrial Cancer

**DOI:** 10.3390/cancers12123860

**Published:** 2020-12-21

**Authors:** Ludwig Kiesel, Christine Eichbaum, Ariane Baumeier, Michael Eichbaum

**Affiliations:** 1 Department of Gynecology and Obstetrics, University of Münster Medical School, Albert-Schweitzer-Campus 1, 48149 Münster, Germany; a_baum19@uni-muenster.de; 2 Department of Gynecology and Obstetrics, University of Frankfurt Medical School, Theodor-Stern-Kai 7, 60596 Frankfurt, Germany; Christine.Eichbaum@kgu.de; 3 Department of Gynecology and Obstetrics, Helios Dr. Horst-Schmidt-Kliniken Wiesbaden, Ludwig-Erhard-Str. 100, 65199 Wiesbaden, Germany

**Keywords:** endometrial cancer, obesity, body mass index, hyperglycemia, proinflammatory microenvironment, metformin, surgery

## Abstract

**Simple Summary:**

Endometrial cancer is the most frequent gynecologic tumor in developed countries. Obesity is an established risk factor for this disease. This work provides an overview of pathophysiological interactions and pathways in obese women initiating tumorigenesis. Furthermore, the clinical impact of adiposity on the treatment of endometrial cancer is discussed as well therapeutic and preventive options.

**Abstract:**

Endometrial cancer (EC) is the most frequently observed malignant gynecologic disease in developed countries. There is a strong association between the established risk factor obesity and the incidence of EC. Furthermore, the rate of women with a body mass index (BMI) > 30 kg/m^2^ is increasing worldwide, correspondingly leading to a higher prevalence of EC. Understanding the adipose tissue as an endocrine organ, elementary pathophysiological pathways of tumorigenesis have been revealed. This includes the fundamental role of hyperglycemia, insulin resistance, and hyperestrogenemia, as well as interactions with a chronic proinflammatory microenvironment. Therapeutic options potentially include metformin or bariatric surgery. Moreover, changes in individual lifestyle such as weight reduction, physical activity, and an awareness of healthy nutrition are effective in preventing the disease.

## 1. Introduction

Increasing body mass index (BMI) is a phenomenon being seen not only in the classical Western world but also in developing countries [1,2]. Indeed, one-third of American women are obese, defined by a BMI > 30 kg/m^2^, with about 7% presenting with severe or morbid obesity (BMI > 40 kg/m^2^) [3]. There is a strong, well-established association between obesity and the development of endometrial cancer (EC) [4,5]. A meta-analysis of the American Institute for Cancer Research recently found that for every increase of five BMI units, there was a 50% increase for the risk of developing EC [6]. As a consequence, the incidence of EC is constantly increasing worldwide [7,8].

However, many women are still not aware of this very individual risk factor [3,9]. Thus, there is a strong demand to improve the common understanding of the relationship between adiposity and EC and to offer therapeutic and behavioral strategies.

This communication provides an overview of the main, known pathophysiological pathways and mechanisms connecting obesity and EC. In addition, implications for therapy and prevention are discussed.

## 2. Adipose-Derived Pathophysiological Mechanisms Linking EC to Adiposity

The link between obesity and EC is a highly complex system consisting of several closely intertwined pathways and mechanisms. Adiposity influences not only the metabolism but is also highly associated with hyperlipidemia, insulin resistance, hyperglycemia, and hyperinsulinemia. Fat tissue and its components have pro- and anti-inflammatory characteristics and function as a source of hormone production, thus playing a key role in endometrial proliferation. Via complex signaling pathways, receptors, and gene expression, fat tissue not only induces proliferation but also invasion and modulation of cancer cells.

### 2.1. Estrogen-Driven Tumorigenesis

**Figure 1:**
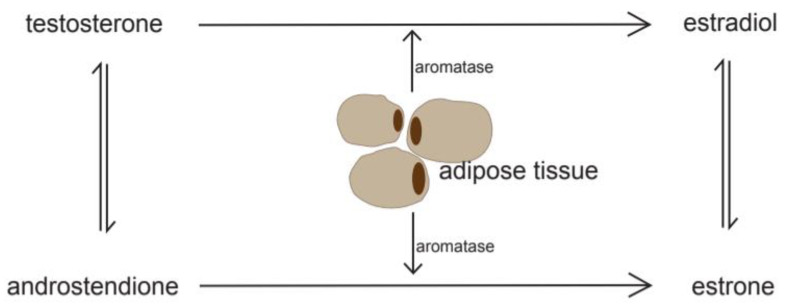
Adipose tissue and steroid conversions.

In premenopausal women, endometrial proliferation due to cyclic estrogen expression of the ovarian tissue is essential for a healthy menstrual cycle. Naturally, estrogen levels decrease in the ovaries in postmenopausal women, and the role of peripheral and adipose tissue in estrogen synthesis gain in importance [8]. Adipose tissue expresses aromatase, an enzyme that catalyzes the endogenous conversion of androgen to estrogen [10]. Thus, with an increase in adipose tissue, estrogen levels increase (Figure 1). Similarly, the amount of sex hormone-binding globulin (SHBG), a hormone that binds and transports estrogen, decreases. Consequently, the level of bioactive estrogen in the circulating bloodstream increases further. In 1996, Potischman et al. showed that an increased risk of EC is directly associated with a high level of circulating, unopposed estrogen and a low plasma level of SHBG [11]. While both estrone and estradiol are linked to an increased risk, the highest risk was observed for unconjugated estradiol [12]. Friedenreich et al. showed in 2020 that the influence of menopause in EC emerges from estrone (*p* = 0.02), estradiol (*p* = 0.006), and, moreover, androstenedione (*p* = 0.01), but not from SHBG or testosterone [13]. The effect attributed to elevated levels of androstenedione might mainly be driven by their conversion to estrogen [14].

The oncogenic signal of estrogen is mediated through estrogen receptors α and β (ERα and ERβ), modulating transcriptional nuclear signaling [15]. In Ishikawa and KLE cell lines, ERα and Eβ promote cell invasion, migration, and proliferation, suggesting an association between receptor overexpression, invasiveness, and metastasis of EC [16]. ERs induce an overstimulation of the phosphatidylinositol 3-kinase (PI3K)/AKT/mammalian target of rapamycin (mTOR) signaling pathway in EC cell lines and thus play a role in epithelial-mesenchymal transition (EMT) [16,17]. In both types of EC, estrogen induces increased levels of phosphorylation of PI3K p85α and activates phosphorylation of mTOR and AKT [16].

Yang et al. recently discovered that estrogen-induced upregulation of prohibitin expression represents an element of endometrial tumorigenesis and the estrogen-ER signaling pathway. By inhibiting ubiquitination, estrogen stabilizes prohibitin, while ERα mediates protein expression. High prohibitin levels are linked to a poor EC prognosis [18].

In 2011, Zhang et al. further investigated the role of fat mass and obesity-associated (FTO) gene as FTO was found to be overexpressed in endometrial tumor tissue in white, non-Hispanic [19], and Chinese women. By activating the PI3K/AKT and MPAK signal pathway, β-estradiol (E2) induced FTO evokes tumor proliferation and invasion [20]. However, E2 also interferes in EC cell invasion and migration via activation of IL-6 pathway and its target genes (Bcl-2, Mcl.1, cyclin D1 and MMP2) [21].

In addition to ERα and ERβ, estrogen also binds to G protein-coupled estrogen receptor (GPER), promoting non-nuclear-regulated apoptosis, migration, and cell growth [22].

Accordingly, the estrogen response causes tumorigenesis by damaging DNA strands, stimulating angiogenesis, and promoting cell proliferation and mutagenesis.

However, already in 1996, Potischman et al. and later Brinton et al. in 2017 reported that high estrogen levels could only partially explain the link between obesity and EC; however, BMI remained a significant hazard factor. Dossus et al. linked two other constituents in addition to steroids to elevated EC risk in postmenopausal women: insulin resistance/metabolic syndrome and inflammation [23].

### 2.2. Adipose Tissue—An Endocrine Organ

In this context, hypertrophied adipose tissue assumes the function of an endocrine organ in human metabolism. It not only secretes estrogen, but also adiponectin, visfatin, resistin, leptin, and tumor necrosis factor-α (TNFα).

**Figure 2:**
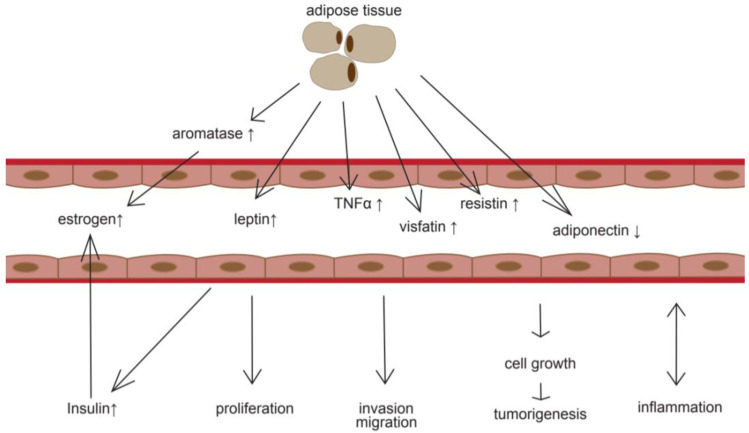
Simplified mechanisms of adipose tissue influence on tumorigenesis.

High serum levels of visfatin were found to be associated with cell proliferation, differentiation, and inhibition of apoptosis in EC, especially in obese patients (BMI > 25) and correlate with tissue differentiation [24] and FIGO EC stage [25]. Likewise, Dallal et al. found high leptin serum levels as a predictive factor for EC risk [26]. Leptin binding to leptin receptors activates JAK2/STAT3, MAPK, and PI3K/Akt signaling pathways and thus modulates cell proliferation. Furthermore, leptin promotes a proinflammatory microenvironment, stimulating cytokines and T-helper 1 cells. This state of chronic inflammation modulating immune responses enhances angiogenesis and growth of cancer cells [27] (Figure 2).

Likewise, adipocytes also produce adiponectin, a protein attributed to both anti-inflammatory and antiproliferative actions [28]. Serum adiponectin level was found to be decreased in women with EC and related to a poor prognosis [29]. Wang et al. were able to show an inverse correlation between adiponectin serum levels, FIGO stage, and tissue differentiation [24]. Furthermore, visfatin/adiponectin ratio and leptin/adiponectin ratio were independently associated with an increased risk for developing EC [24,30]. Although the association of hypoadiponectinemia and EC risk was shown to be independent of BMI, adiponectin secretion decreases in particular in visceral adipose tissue with increasing adiposity [31]. Weyer et al. suggested, however, that low serum levels of adiponectin were associated with hyperinsulinemia and insulin resistance independent of the degree of adiposity [32].

### 2.3. The Link between Estrogen, Insulin Resistance, and Cancer

Decrease in adiponectin and release of leptin, interleukin-6, TNFα and free fatty acids influence the response of certain body tissue to insulin [33]. Thus, adipokines, interleukins and TNFα induce insulin resistance, while adiponectin can be understood as an endogenous insulin sensitizer.

Hyperinsulinemia is considered a comorbidity of obesity and represents an estrogen-independent endometrial carcinoma risk factor [34]. Insulin and IGF-1 have been found to be associated with angiogenesis [35] and differentiation [36]. By reducing levels of IGF-binding proteins-1 and -2, insulin increases levels of IGF-1 and IGF-2 in the serum. IGF-1 inhibits cell apoptosis and promotes cell proliferation via the MAPK and PI3K/mTOR signaling pathway [37]. High levels of IGF-1 and insulin then depress SHBG synthesis again in the liver and induce steroidogenesis, increasing bioavailable estrogen levels.

Analogous to hypoadiponectemia, hyperinsulinemia was found to be a risk factor of EC independently of BMI [38,39]. Another comorbidity of adiposity is hypercholesterolemia, which recently was linked to cancer growth as the potentially missing link between obesity and cancer. As a cholesterol metabolite, 27-hydroxycholesterol (27HC) functions as an endogenous selective ER modulator (SERM). Serum concentrations of ER increase with age and with serum cholesterol levels [40].

Obesity constitutes a risk factor for EC; therefore, adipose tissue must be seen as a highly complex, multifactorial component in tumorigenesis. Adipose tissue functions as an endocrine organ; thus, obesity results in a dysregulated secretion of proinflammatory cytokines, adipocytes, dysfunction, and infiltration of immune cells, inducing damaged DNA strands, angiogenesis, cell proliferation, and mutagenesis. Weihe et al. found that this association started during childhood and adolescence, which increases cancer risk in adulthood [41]. However, certain risk factors, such as hyperinsulinemia, insulin resistance, and hypoadiponectinemia, were shown to be independent of BMI, partially explaining mechanisms of endometrial carcinogenesis in nonobese women. Cancer-inducing interactions among all involved components are still not fully understood. Nonetheless, these risk factors, seen as comorbidities, are related to adiposity, underscoring the relevance of obesity in endometrial tumorigenesis.

## 3. Clinical Management and Treatment

### 3.1. Metformin

The biguanide metformin is commonly used as antidiabetic drug, mainly as standard medication for patients suffering from type 2 diabetes mellitus. However, preclinical studies also confirmed an antineoplastic activity of this molecule. On the one hand, metformin influences several cellular growth and proliferation signaling pathways, in particular the PI3K/AKT/mTOR or PI3K/AKT/MDM2 pathway [42,43]. On the other hand, there is evidence that metformin inhibits EMT, for example, by increasing E-cadherin expression [44]. Recent reports also demonstrated that metformin inhibited PD-L1 in an EC cell line model and thereby can suppress cancer cell growth [45].

There is increasing evidence that these antineoplastic effects can be used to prevent and treat EC [46]. In 2017, Meireles and colleagues published a systematic review of 19 relatively heterogeneous studies showing that metformin reversed atypic endometrial hyperplasia [46,47]. In addition, another systematic review demonstrated that metformin treatment was associated with a significantly decreased mortality among postmenopausal EC patients [48]. In contrast to these reports, in a systematic review of seven studies in 2019, Chu and colleagues could not confirm a risk reduction for EC by metformin [49]. Nevertheless, the same authors proved that relapse in EC patients was significantly reduced and overall survival significantly prolonged (HR = 0.47, 95% CI 0.33–0.67, *p* < 0.05).

Whether metformin can be directly used as an antineoplastic agent for the treatment of EC is currently still the topic of controversial debate [46,50,51,52,53,54,55].

### 3.2. Obesity and Its Consequences for Surgical Treatment

Total laparoscopic hysterectomy with bilateral salpingoophorectomy is a routine surgical procedure with curative intent in patients with early-stage EC [56]. One advantage of minimally invasive surgery in obese patients is that major perioperative morbidity associated with wound breakdown of larger mid-line incision scars can be avoided [57]. Furthermore, significantly less blood loss is attributed to laparoscopic interventions than to open surgery and patients can be mobilized faster during the postoperative period. Thus, the risk of thrombosis can be reduced and reconvalescence can be improved [58]. Gambacorti-Passerini and colleagues confirmed these findings in an observational study on 83 patients, pointing out that laparoscopic surgery can be safely offered to obese patients with EC [59].

However, the Trendelenburg position is mandatory for laparoscopic hysterectomy. A recently published systematic review and meta-analysis of 51 observational studies on 10,800 patients found a conversion rate of 7% for morbidly obese patients undergoing laparoscopic surgery, whereas in 31% of these cases, the reason for conversion was an intolerance of Trendelenburg position [60]. In that study, the authors also found that robotic surgery was better able to avoid conversions.

Nevertheless, there is consensus that adiposity is associated with a highly increased rate of perioperative complications and morbidity following laparotomy, including greater blood loss, wound infections, secondary wound breakdown, thrombosis, and hospital stay [61]. Finally, in cases of extreme morbid obesity, any surgical access might be impossible, either because anesthesiological surveillance of the patient is insufficient or because there is no surgical route to the surgically relevant structures in the pelvis mainly due to the intra-abdominal adiposity.

### 3.3. Possible Complications of Chemotherapy and Radiotherapy Due to Obesity

There is some evidence that obese patients show differences in pharmacokinetics and metabolic dysregulation compared to non-obese patients [62]. However, there is very little evidence that these differences lead to increased toxicity among obese patients [63]. Moreover, the main risk factor for obese patients under chemotherapy can be seen in the fact that the treatment might be underdosed. Fearing potential—but not really proven—toxicities, physicians still tend to cap the maximum dosage of standard chemotherapy regimens at 2.0 m^2^ body surface area (BSA) instead of applying the full standard dosage. In 2012, a special ASCO guideline focused on this issue, after stating that in 2012 up to 40% of all obese cancer patients were treated with suboptimally dosed chemotherapy regimens [64]. In line with these data, in 2009 Schwartz et al. reported on a retrospective study on 59 obese patients with a BSA > 2.0 m^2^ receiving adjuvant chemotherapy with carboplatin and paclitaxel for endometrial or ovarian cancer [65]. A total of 50 patients were treated with standard paclitaxel dosages according to the actual body weight, whereas in nine patients, the paclitaxel dosages were capped at a BSA of 2.0 m^2^. Interestingly, the authors did not find any significant differences between both groups in rates of toxicity or dose modification. Thus, they concluded that any empiric dose reduction is unnecessary and may lead to suboptimal cancer treatment.

Radiotherapy is also a cornerstone of standard treatment of primary endometrial cancer. In parallel to the chemotherapy-related effects discussed above, there is also a lack of evidence regarding the effects of obesity on potential radiotherapy complications. In 2017, Smits and colleagues reported the result of a retrospective cohort study on 159 women diagnosed with endometrial cancer who received radiotherapy as part of their general primary treatment including external beam radiotherapy as well as vaginal brachytherapy [66]. In the analyzed cohort, 63 women had a BMI < 30 kg/m^2^ and 47 women were obese. As main result of this study, BMI was not associated with the incidence of acute and late radiation toxicities in the different radiotherapy groups and there were no differences in individual complication between the BMI groups.

## 4. Weight Reduction and Its Value as Prevention Measure

### 4.1. Lifestyle

There is increasing evidence that intentional weight loss significantly reduces incidence and mortality of EC in obese patients. The Women’s Health Initiative Study (WHI), for example, demonstrated that an intentional weight loss of >5% was associated with a significantly reduced EC risk [46,67,68]. This was also confirmed in a systematic review and meta-analysis by Zhang et al. in 2019. The authors reported that intentional weight loss and maintaining a stable, healthy weight were associated with a significantly lower risk of EC (RR range 0.61–0.96) [69].

The question of weight loss is relevant as Haggerty and colleagues pointed out in 2017. They conducted a survey among patients with stage I EC and found that 59% of the patients reported great interest in using weight loss as a preventive method against recurrence [3].

Intentional weight loss can, on the one hand, be achieved by maintaining a healthy and effective diet to reasonably reduce weight. On the other hand, increasing physical activity, for example, by exercise is also a potential method of losing weight. There is consensus that physical activity is beneficial for reducing individual cancer risk [70,71,72]. In particular, the risk for developing EC can be positively affected [73,74].

### 4.2. Bariatric Surgery

Bariatric surgery interventions can lead to substantial weight loss. In 2007, Sjostrom and colleagues reported the results of the Swedish Obese Subjects (SOS) study [75]. In this trial, 1420 obese women who underwent bariatric surgery were observed and compared to a control group that received “conventional”, conservative obesity treatment such as diet, increase in physical activity, etc. In this trial, bariatric surgery led to a significantly reduced incidence of EC (HR = 0.56, *p* = 0.014) after a median follow-up period of 18.2 years [46,76]. These findings were recently confirmed and underscored by data from a systematic review published by Winder et al. [77]. The authors analyzed the results of five prospective trials comparing women who underwent bariatric surgery with a control group with respect to their risk of developing EC. As a key result of this study, a significant risk reduction for EC was found for the bariatric surgery group (OR = 0.317, 95% CI = 0.161–0.627).

The pathophysiological mechanism underlying the effects of bariatric surgery is still not fully understood. One possible pathway of action might be the decrease in glucose levels as well as beneficial influences from insulin levels and associated cytokines. In addition, bariatric surgery was reported to decrease serum levels of proinflammatory cytokines [78].

### 4.3. Implications of Weight Loss after Diagnosis of Endometrial Cancer

In contrast to the presented evidence for the beneficial effects of weight reduction in order to prevent the origination of endometrial cancer, there is little information on the effects of weight loss after diagnosis of the disease. In 2012 in a retrospective study El-Safadi and co-authors analyzed 705 patients with endometrial cancer under follow-up. The records of all patients were reviewed for body weight after the diagnosis of EC and during follow-up [79]. Finally, the recurrence-free interval was determined. Interestingly, in this study a moderate weight gain of ≤1 kg/m^2^ was associated with the best prognosis among these patients. In contrast, patients who showed a weight loss, had a worse prognosis. However, the authors did not differentiate between intentional and non-intentional weight-loss. In addition, the statistical power of this retrospective analysis was low. Nevertheless, currently there is no strong evidence given that once an endometrial cancer has been originated weight reduction would lead to definite improvement of outcome.

## 5. Conclusions

Endometrial cancer (EC) is the most frequently observed malignant gynecologic disease in developed countries.

Obesity presents one risk factor in tumorigenesis of EC as adipose tissue can been understood as an endocrine organ resulting in a disbalanced levels of estrogen, pro- and anti-inflammatory cytokines and adipocytes. This imbalance leads to a dysregulation of insulin resistance and hyperglycemia, to an infiltration of immune cells, inducing damaged DNA strands, angiogenesis, increased cell proliferation rates and mutagenesis. The interactions of all these components in tumorigenesis are still not fully understood. Understanding these risk factors as comorbidities related to adiposity, they emphasize the relevance of obesity in endometrial tumorigenesis.

There is broad evidence that the antidiabetic agent metformin has antineoplastic properties and can be used in the prevention and treatment of endometrial cancer.

Obesity has a major impact on surgery for EC and is also of relevance for chemotherapy and radiotherapy.

Intentional weight loss as well as bariatric surgery are effective to reduce the risk for the origination of endometrial cancer.

## References

[1] Afshin A., Forouzanfar M.H., Reitsma M.B., Sur P., Estep K., Lee A., Marczak L., Mokdad A.H., Moradi-Lakeh M., GBD 2015 Obesity Collaborators (2017). Health effects of overweight and obesity in 195 countries over 25 years. N. Engl. J. Med..

[2] Friedenreich C.M., Ryder-Burbidge C., McNeil J. (2020). Physical activity, obesity and sedentary behavior in cancer etiology: Epidemiologic evidence and biologic mechanisms. Mol. Oncol..

[3] Haggerty A.F., Sarwer D.B., Schmitz K.H., Ko E.M., Allison K.C., Chu C.S. (2017). Obesity and Endometrial Cancer: A Lack of Knowledge but Opportunity for Intervention. Nutr. Cancer.

[4] Reeves G.K., Pirie K., Beral V., Green J., Spencer E., Bull D. (2007). Cancer incidence and mortality in relation to body mass index in the Million Women Study: Cohort study. BMJ.

[5] Onstad M.A., Schmandt R.E., Lu K.H. (2016). Addressing the Role of Obesity in Endometrial Cancer Risk, Prevention, and Treatment. J. Clin. Oncol..

[6] (2013). World Cancer Research Fund/American Institute for Cancer Research: Continuous Update Project Report. Food: Nutrition, Physical Activity, and the Prevention of Endometrial Cancer. http://www.dietandcancerreport.org.

[7] Bray F., Ferlay J., Soerjomataram I., Siegel R.L., Torre L.A., Jemal A. (2018). Global cancer statistics 2018: GLOBOCAN estimates of incidence and mortality worldwide for 36 cancers in 185 countries. CA Cancer J. Clin..

[8] Moore K., Brewer M.A. (2017). Endometrial Cancer: Is This a New Disease?. Am. Soc. Clin. Oncol. Educ. Book.

[9] Connor E.V., Raker C.A., Clark M.A., Stuckey A.R. (2017). Obesity risk awareness in women with endometrial cancer. Arch. Gynecol. Obstet..

[10] Simpson E.R., Mahendroo M.S., Means G.D., Kilgore M.W., Hinshelwood M.M., Graham-Lorence S., Amarneh B., Ito Y., Fisher C.R., Michael M.D. (1994). Aromatase Cytochrome P450, The Enzyme Responsible for Estrogen. Biosynthesis. Endocr. Rev..

[11] Potischman N., Hoover R.N., Brinton L.A., Siiteri P., Dorgan J.F., Swanson C.A., Berman M.L., Mortel R., Twiggs L.B., Barrett R.J. (1996). Case—Control Study of Endogenous Steroid Hormones and Endometrial Cancer. J. Natl. Cancer Inst..

[12] Brinton L.A., Trabert B., Anderson G.L., Falk R.T., Felix A.S., Fuhrman B.J., Gass M.L., Kuller L.H., Pfeiffer R.M., Rohan T.E. (2016). Serum Estrogens and Estrogen Metabolites and Endometrial Cancer Risk among Postmenopausal Women. Cancer Epidemiol. Biomark. Prev..

[13] Friedenreich C.M., Derksen J., Speidel T., Brenner D.R., Heer E., Courneya K.S., Cook L.S. (2020). Case—control study of endogenous sex steroid hormones and risk of endometrial cancer. Cancer Causes Control.

[14] Lukanova A., Lundin E., Micheli A., Arslan A., Ferrari P., Rinaldi S., Krogh V., Lenner P., Shore R.E., Biessy C. (2004). Circulating levels of sex steroid hormones and risk of endometrial cancer in postmenopausal women. Int. J. Cancer.

[15] Rodriguez A.C., Blanchard Z., Maurer K.A., Gertz J. (2019). Estrogen Signaling in Endometrial Cancer: A Key Oncogenic Pathway with Several Open Questions. Horm. Cancer.

[16] Hou X., Zhao M., Wang T., Zhang G. (2014). Upregulation of estrogen receptor mediates migration, invasion and proliferation of endometrial carcinoma cells by regulating the PI3K/AKT/mTOR pathway. Oncol. Rep..

[17] Guo R.X., Wei L.H., Tu Z., Sun P.M., Wang J.L., Zhao D., Li X.P., Tang J.M. (2006). 17 beta-estradiol activates PI3K/Akt signaling pathway by estrogen receptor (ER)-dependent and ER-independent mechanisms in endometrial cancer cells. J. Steroid Biochem. Mol. Biol..

[18] Yang B., Chen R., Liang X., Shi J., Wu X., Zhang Z., Chen X. (2019). Estrogen Enhances Endometrial Cancer Cells Proliferation by Upregulation of Prohibitin. J. Cancer.

[19] Lurie G., Gaudet M.M., Spurdle A.B., Carney M.E., Wilkens L.R., Yang H.P., Weiss N.S., Webb P.M., Thompson P.J., Terada K. (2011). The obesity-associated polymorphisms FTO rs9939609 and MC4R rs17782313 and endometrial cancer risk in non-Hispanic white women. PLoS ONE.

[20] Zhang Z., Zhou D., Lai Y., Liu Y., Tao X., Wang Q., Zhao G., Gu H., Liao H., Zhu Y. (2012). Estrogen induces endometrial cancer cell proliferation and invasion by regulating the fat mass and obesity-associated gene via PI3K/AKT and MAPK signaling pathways. Cancer Lett..

[21] Che Q., Xiao X., Xu J., Liu M., Lu Y., Liu S., Dong X. (2019). 17β-Estradiol promotes endometrial cancer proliferation and invasion through IL-6 pathway. Endocr. Connect..

[22] Prossnitz E.R., Barton M. (2011). The G-protein-coupled estrogen receptor GPER in health and disease. Nat. Rev. Endocr..

[23] Dossus L., Lukanova A., Rinaldi S., Allen N., Cust A.E., Becker S., Tjonneland A., Hansen L., Overvad K., Chabbert-Buffet N. (2013). Hormonal, metabolic, and inflammatory profiles and endometrial cancer risk within the EPIC cohort--a factor analysis. Am. J. Epidemiol..

[24] Wang Z., Gao S., Sun C., Li J., Gao W., Yu L. (2019). Clinical significance of serum adiponectin and visfatin levels in endometrial cancer. Int. J. Gynaecol. Obstet..

[25] Tian W., Zhu Y., Wang Y., Teng F., Zhang H., Liu G., Ma X., Sun D., Rohan T., Xue F. (2013). Visfatin, a potential biomarker and prognostic factor for endometrial cancer. Gynecol. Oncol..

[26] Dallal C.M., Brinton L.A., Bauer D.C., Buist D.S., Cauley J.A., Hue T.F., Lacroix A., Tice J.A., Chia V.M., Falk R. (2013). Obesity-related hormones and endometrial cancer among postmenopausal women: A nested case—control study within the B~FIT cohort. Endocr.-Relat. Cancer.

[27] Atoum M.F., Alzoughool F., Al-Hourani H. (2020). Linkage Between Obesity Leptin and Breast Cancer. Breast Cancer Basic Clin. Res..

[28] Tumminia A., Vinciguerra F., Parisi M., Graziano M., Sciacca L., Baratta R., Frittitta L. (2019). Adipose Tissue, Obesity and Adiponectin: Role in Endocrine Cancer Risk. Int. J. Mol. Sci..

[29] Soliman P.T., Wu D., Tortolero-Luna G., Schmeler K.M., Slomovitz B.M., Bray M.S., Gershenson D.M., Lu K.H. (2006). Association between adiponectin, insulin resistance, and endometrial cancer. Cancer.

[30] Ashizawa N., Yahata T., Quan J., Adachi S., Yoshihara K., Tanaka K. (2010). Serum leptin-adiponectin ratio and endometrial cancer risk in postmenopausal female subjects. Gynecol. Oncol..

[31] Reneau J., Goldblatt M., Gould J., Kindel T., Kastenmeier A., Higgins R., Rengel L.R., Schoyer K., James R., Obi B. (2018). Effect of adiposity on tissue-specific adiponectin secretion. PLoS ONE.

[32] Weyer C., Funahashi T., Tanaka S., Hotta K., Matsuzawa Y., Pratley R.E., Tataranni P.A. (2001). Hypoadiponectinemia in obesity and type 2 diabetes: Close association with insulin resistance and hyperinsulinemia. J. Clin. Endocrinol. Metab..

[33] Wajchenberg B.L. (2000). Subcutaneous and visceral adipose tissue: Their relation to the metabolic syndrome. Endocr. Rev..

[34] Gunter M.J., Hoover D.R., Yu H., Wassertheil-Smoller S., Manson J.E., Li J., Harris T.G., Rohan T.E., Xue X., Ho G.Y. (2008). A prospective evaluation of insulin and insulin-like growth factor-I as risk factors for endometrial cancer. Cancer Epidemiol. Biomark. Prev..

[35] Kluge A., Zimmermann R., Münkel B., Mohri M., Sack S., Schaper J., Schaper W. (1995). Insulin-like growth factor I is involved in inflammation linked angiogenic processes after microembolisation in porcine heart. Cardiovasc. Res..

[36] Khandwala H.M., McCutcheon I.E., Flyvbjerg A., Friend K.E. (2000). The effects of insulin-like growth factors on tumorigenesis and neoplastic growth. Endocr. Rev..

[37] Vrachnis N., Iavazzo C., Iliodromiti Z., Sifakis S., Alexandrou A., Siristatidis C., Grigoriadis C., Botsis D., Creatsas G. (2016). Diabetes mellitus and gynecologic cancer: Molecular mechanisms, epidemiological, clinical and prognostic perspectives. Arch. Gynecol. Obstet..

[38] Nead K.T., Sharp S.J., Thompson D.J., Painter J.N., Savage D.B., Semple R.K., Barker A., Perry J.R., Attia J., Australian National Endometrial Cancer Study Group (ANECS) (2015). Evidence of a Causal Association Between Insulinemia and Endometrial Cancer: A Mendelian Randomization Analysis. J. Nat. Cancer Inst..

[39] Troisi R., Potischman N., Hoover R.N., Siiteri P., Brinton L.A. (1997). Insulin and Endometrial Cancer. Am. J. Epidemiol..

[40] Asghari A., Umetani M. (2020). Obesity and Cancer: 27-Hydroxycholesterol, the Missing Link. Int. J. Mol. Sci..

[41] Weihe P., Spielmann J., Kielstein H., Henning-Klusmann J., Weihrauch-Blüher S. (2020). Childhood Obesity and Cancer Risk in Adulthood. Curr. Obes. Rep..

[42] Zhao Y., Sun H., Feng M., Zhao J., Zhao X., Wan Q., Cai D. (2018). Metformin is associated with reduced cell proliferation in human endometrial cancer by inbibiting PI3K/AKT/mTOR signaling. Gynecol. Endocrinol..

[43] Qiang P., Shao Y., Sun Y.P., Zhang J., Chen L.J. (2019). Metformin inhibits proliferation and migration of endometrial cancer cells through regulating PI3K/AKT/MDM2 pathway. Eur. Rev. Med. Pharmacol. Sci..

[44] Liu Z., Qi S., Zhao X., Li M., Ding S., Lu J., Zhang H. (2016). Metformin inhibits 17β-estradiol-induced epithelial-to-mesenchymal transition via βKlotho-related ERK1/2 signaling and AMPKα signaling in endometrial adenocarcinoma cells. Oncotargets.

[45] Xue J., Li L., Li N., Li F., Qin X., Li T., Liu M. (2019). Metformin suppresses cancer cell growth i n endometrial carcinoma by inhibiting PD-L1. Eur. J. Pharmacol..

[46] Byrne F.L., Martin A.R., Kosasih M., Caruana B.T., Farrell R. (2020). The Role of Hyperglycemia in Endometrial Cancer Pathogenesis. Cancers.

[47] Meireles C.G., Pereira S.A., Valadares L.P., Rego D.F., Simeoni L.A., Guerra E.N.S., Lofrano-Porto A. (2017). Effects of metformin on endometrial cancer: Systematic review and meta-analysis. Gynecol. Oncol..

[48] Perez-Lopez F.R., Pasupuleti V., Gianuzzi X., Palma-Ardiles G., Hernandez-Fernandez W., Adrian V.H. (2017). Systematic review and meta-analysis of the effect of metformin treatment on overall mortality rates in women with endometrial cancer and type 2 diabetes mellitus. Maturitas.

[49] Chu D., Wu J., Wang K., Zhao M., Wang C., Li L., Guo R. (2018). Effect of metformin use on the risk and prognosis of endometrial cancer: A systematic review and meta-analysis. BMC Cancer.

[50] Nevadunsky N.S., Van Arsdale A., Strickler H.D., Moadel A., Kaur G., Frimer M., Conroy E., Goldberg G.L., Einstein M.H. (2014). Metformin use and endometrial cancer survival. Gynecol. Oncol..

[51] Ko E.M., Walter P., Jackson A., Clark L., Franasiak J., Bolac C., Havrilesky L.J., Secord A.A., Moore D.T., Gehrig P.A. (2014). Metformin is associated with improved survival in endometrial cancer. Gynecol. Oncol..

[52] Al Hilli M.M., Bakkum-Gamez J.N., Mariani A., Cliby W.A., Mc Gree M.E., Weaver A.L., Dowdy S.C., Podratz K.C. (2016). The effect of diabetes and metformin on clinical outcomes is negligible in risk-adjusted endometrial cancer cohorts. Gynecol. Oncol..

[53] Lemanska A., Zaborowski M., Spaczynski M., Nowak-Markwitz E. (2015). Do endometrial cancer patients benefit from metformin intake?. Ginekol. Pol..

[54] Seebacher V., Bergmeister B., Grimm C., Koelbl H., Reinthaller A., Polterauer S. (2016). The prognostic role of metformin in patients with endometrial cancer: A retrospective study. Eur. J. Obstet. Gynecol. Reprod. Biol..

[55] Arima R., Hautakoski A., Marttila M., Arffman M., Sund R., Ilanne-Parikka P., Kangaskokko J., Hinkula M., Puistola U., Läärä E. (2017). Cause-specific mortality in endometrioid endometrial cancer patients with type 2 diabetes using metformin or other types of antidiabetic medication. Gynecol. Oncol..

[56] Janda M., Gebski V., Davies L.C., Forder P., Brand A., Hogg R., Jobling T.W., Land R., Manolitsas T., Nascimento M. (2017). Effect of Total Laparoscopic Hysterectomy vs. Total Abdominal Hysterectomy on Disease-Free Survival Among Women with Stage I Endometrial Cancer: A Randomized Clinical Trial. JAMA.

[57] Mahdi H., Jernigan A.M., Aljebori Q., Lockhart D., Moslemi-Kebria M. (2015). The impact of obesity on the 30-day morbidity and mortality after surgery for endometrial cancer. J. Minim. Invasive Gynecol..

[58] Al Sawah E., Salemi J.L., Hoffman M., Imudia A.N., Mikhail E. (2018). Association between Obesity, Surgical Route, and Perioperative Outcomes in Patients with Uterine Cancer. Minim. Invasive Surg..

[59] Gambacorti-Passerini Z.M., López-De la Manzanara Cano C., Pérez Parra C., Cespedes Casas M.C., Sánchez Hipólito L., Martín Francisco C., Muñoz-Rodríguez J.R. (2019). Obesity in Patients with Endometrial Cancer: May It Affect the Surgical Outcomes of Laparoscopic Approach?. Obes. Surg..

[60] Cusimano M.C., Simpson A.N., Dossa F., Liani V., Kaur Y., Acuna S.A., Robertson D., Satkunaratnam A., Bernardini M.Q., Ferguson S.E. (2017). Laparoscopic and robotic hysterectomy in endometrial cancer patients with obesity: A systematic review and meta-analysis of conversions and complications. Arch. Gynecol. Obstet..

[61] Orekoya O., Samson M.E., Trivedi T., Vyas S., Steck S.E. (2016). The impact of obesity on surgical outcome in endometrial cancer patients: A systematic review. J. Gynecol. Surg..

[62] Horowitz N.S., Wright A.A. (2015). Impact of obesity on chemotherapy management and outcomes in women with gynecologic malignancies. Gynecol. Oncol..

[63] Ottaiano A., De Divitiis C., Capozzi M., Avallone A., Pisano C., Pignata S., Tafuto S. (2018). Obesity and Cancer: Biological Links and Treatment Implications. Curr. Cancer Drug Targ..

[64] Griggs J.J., Mangu P.B., Anderson H., Balaban E.P., Dignam J.J., Hryniuk W.M., Morrison V.A., Pini T.M., Runowicz C.D., Rosner G.L. (2012). American Society of Clinical Oncology. Appropriate chemotherapy dosing for obese adult patients with cancer: American Society of Clinical Oncology clinical practice guideline. J. Clin. Oncol..

[65] Schwartz J., Toste B., Dizon D.S. (2009). Chemotherapy toxicity in gynecologic cancer patients with a body surface area (BSA)>2 m2. Gynecol. Oncol..

[66] Smits A., McGrane J., Lopes A., Kent E., Bekkers R., Massuger L., Simpson N., Galaal K. (2017). Radiation-related toxicities and outcomes in endometrial cancer: Are obese women at a disadvantage?. Int. J. Clin. Oncol..

[67] Luo J., Chlebowski R.T., Hendryx M., Rohan T., Wactawski-Wende J., Thomson C.A., Felix A.S., Chen C., Barrington W., Coday M. (2017). Intentional weight loss and endometrial cancer risk. J. Clin. Oncol..

[68] Luo J., Hendryx M., Manson J.E., Figueiredo J.C., LeBlanc E.S., Barrington W., Rohan T.E., Howard B.V., Reding K., Ho G.Y. (2019). Intentional weight loss and obesity-related cancer risk. JNCI Cancer Spectr..

[69] Zhang X., Rhoades J., Caan B.J., Cohn D.E., Salani R., Noria S., Suarez A.A., Paskett E.D., Felix A.S. (2019). Intentional weight loss, weight cycling, and endometrial cancer risk: A systematic review and meta-analysis. Int. J. Gynecol. Cancer.

[70] Moore S.C., Lee I., Weiderpass E., Campbell P.T., Sampson J.N., Kitahara C.M., Keadle S.K., Arem H., Berrington de Gonzalez A., Hartge P. (2019). Association of leisure-time physical activity with risk of 26 types of cancer in 1.44 million adults. Complement Ther. Med..

[71] Lugo D., Pulido A.L., Mihos C.G., Issa O., Cusnir M., Horvath S.A., Lin J., Santana O. (2016). The effects of physical activity on cancer prevention, treatment and prognosis: A review of the literature. JAMA Intern. Med..

[72] Kerr J., Anderson C., Lippman S.M. (2017). Physical activity, sedentary behaviour, diet, and cancer: An update and emerging new evidence. Lancet Oncol..

[73] Koutoukidis D.A., Knobf M.T., Lanceley A. (2015). Obesity, diet, physical activity, and health-related quality of life in endometrial cancer survivors. Nutr. Rev..

[74] Demark-Wahnefried W., Morey M.C., Sloane R., Snyder D.C., Miller P.E., Hartman T.J., Cohenet H.J. (2012). Reach out to enhance wellness home-based diet-exercise intervention promotes reproducible and sustainable long-term improvements in health behaviors, body weight, and physical functioning in older, overweight/obese cancer survivors. J. Clin. Oncol..

[75] Sjostrom L., Narbro K., Sjostrom C.D., Karason K., Larsson B., Wedel H., Lystig T., Sullivan M., Bouchard C., Carlsson B. (2007). Effects of bariatric surgery on mortality in Swedish obese subjects. N. Engl. J. Med..

[76] Anveden Å., Taube M., Peltonen M., Jacobson P., Andersson-Assarsson J.C., Sjöholm K., Svensson P.-A., Carlsson L.M.S. (2017). Long-term incidence of female-specific cancer after bariatric surgery or usual care in the Swedish Obese Subjects Study. Gynecol. Oncol..

[77] Winder A.A., Kularatna M., MacCormick A.D. (2018). Does bariatric surgery affect the incidence of endometrial cancer development? A systematic review. Obes. Surg..

[78] Izquierdo A.G., Carreira M.C., Rodriguez-Carnero G., Fernandez-Quintela A., Sueiro A.M., Martinez-Olmos M.A., Guzman G., De Luis D., Pinhel M.A.S., Nicoletti C.F. (2020). Weight loss normalizes enhanced expression of the oncogene survivin in visceral adipose tissue and blood leukocytes from individuals with obesity. Int. J. Obes..

[79] El-Safadi S., Sauerbier A., Hackethal A., Münstedt K. (2012). Body weight changes after the diagnosis of endometrial cancer and their influences on disease-related prognosis. Arch. Gynecol. Obstet..

